# Dysmenorrhea and Adolescent Mental Health: A School‐Based Cross‐Sectional Study

**DOI:** 10.1111/1471-0528.18187

**Published:** 2025-04-24

**Authors:** Pietro Gambadauro, Gergö Hadlaczky, Danuta Wasserman, Vladimir Carli

**Affiliations:** ^1^ Department of Learning, Informatics, Management and Ethics Karolinska Institutet Stockholm Sweden; ^2^ Department of Women's and Children's Health Uppsala University Uppsala Sweden; ^3^ Stockholm Health Care Services Stockholm Sweden

**Keywords:** adolescence, anxiety, chronic pain, depression, dysmenorrhea, menarche, menstrual health, mental health, self‐injurious behaviour, suicidal ideation

## Abstract

**Objective:**

While active monitoring of adolescent menstrual and mental health is advocated, research on their possible bidirectional relationship is limited. This study examines the association between adolescent dysmenorrhea and psychological symptoms.

**Design:**

Cross‐sectional study.

**Setting:**

116 schools in Stockholm, Sweden.

**Sample:**

1054 postmenarchal school girls (mean age 14.1 ± 0.7) randomly sampled from a population of 10 299 lower‐secondary pupils in a school‐based project.

**Methods:**

A self‐report health survey assessed psychological symptoms using validated instruments. A multiple‐choice item identified dysmenorrhea (menstrual pain affecting everyday life) and severe dysmenorrhea (dysmenorrhea that is hard to cope with).

**Main Outcome Measures:**

Prevalence of dysmenorrhea and severe dysmenorrhea in girls with and without symptoms of depression (Beck's Depression Inventory‐II score ≥ 20), anxiety (Generalised Anxiety Disorder 7‐Item Scale score ≥ 10), self‐injury (≥ 3 instances on a modified Deliberate Self‐harm Inventory), and suicide ideation (recent serious thoughts/plans on the Paykel Suicide Scale).

**Results:**

Overall, 55.1% reported dysmenorrhea while 11.7% reported severe dysmenorrhea. Prevalence was 29%–34% higher among girls with psychological symptoms compared to those without. Severe dysmenorrhea was significantly more frequent among girls with any symptom (prevalence ratio [PR] 2.25; 95% CI 1.61, 3.13), depression (PR 2.60; 95% CI 1.86, 3.63), anxiety (PR 2.89; 95% CI 2.09, 4.00), self‐injury (PR 1.87; 95% CI 1.29, 2.71), and suicide ideation (PR 1.75; 95% CI 1.18, 2.58) compared to girls without the same manifestations. These findings were consistent after adjustments for age, age of menarche, country of birth, and hormonal contraception.

**Conclusions:**

These findings emphasise the need for integrated approaches to adolescent menstrual and mental health care.

## 1 Introduction

Dysmenorrhea, or painful menstruation, is the most common menstrual symptom and is particularly frequent among adolescents [1, 2]. Prevalence above 70% is often reported during late adolescence both internationally and in Sweden [2, 3, 4, 5]. The origin of adolescent menstrual pain is commonly attributed to a local inflammatory response that occurs during menstruation (primary dysmenorrhea) but can sometimes be related to specific disorders such as endometriosis (secondary dysmenorrhea) [6, 7]. Regardless of etiological background, unmanaged dysmenorrhea can have profound consequences on health and quality of life. In addition to direct physical suffering, young women with dysmenorrhea have reduced psychological well‐being and often miss school, sports, or other social activities [2, 4].

Adolescent mental ill health is also a significant public health challenge globally [8]. Manifestations such as depression, anxiety, non‐suicidal self‐injury, and suicidal ideation are common among adolescents, with girls typically experiencing more symptoms than boys [9, 10, 11]. In Sweden and other Western countries, the incidence of psychiatric diagnoses and referrals to mental health services is increasing, particularly among younger women [12, 13]. Interestingly, differences in mental health between girls and boys emerge during adolescence [14]. Prepubertal girls and boys, for instance, have similar rates of depression, whereas female predominance emerges in mid‐puberty and seems to correlate with pubertal status, rather than age or pubertal timing [15, 16].

Recently, separate calls have been made for active monitoring of adolescent menstrual and mental health [6, 8], but very few studies on possible interrelationships are available [17]. Chronic pain is indeed a common physical complaint among people with psychological disorders [18, 19]. However, adolescent dysmenorrhea is overlooked in pain research as it is often normalised in social contexts [20]. More efforts are therefore needed to identify high‐risk groups and inform successful management strategies.

The aim of this study was to evaluate the association of dysmenorrhea with mental ill health in a large school‐based sample of Swedish postmenarchal adolescents. We hypothesised that dysmenorrhea and severe dysmenorrhea would be more frequent among girls with significant levels of depression, anxiety, non‐suicidal self‐injury, or suicidal ideation, compared to peers without the same psychopathological manifestations.

## 2 Methods

### 2.1 Design and Study Population

Between 2016 and 2019, a mental health promotion initiative called YAM (Youth Aware of Mental health) [21] was implemented by Stockholm Region and Karolinska Institutet in 116 schools located in 24 out of 26 municipalities in Stockholm, Sweden. Within the project, 10 299 volunteering pupils from lower secondary classes (median age 14; IQR 13–14; 5024 girls) completed a baseline self‐report health survey, including mental health questionnaires, on digital mobile devices during a single classroom session. An additional questionnaire including items on menarche and menstrual health was randomly delivered to one‐third of the initial female population (*n* = 1644) [5]. Postmenarchal girls who completed the mental health questionnaires were eligible for the present study, whereas those with missing data on dysmenorrhea were excluded.

### 2.2 Mental Health

Psychopathological symptoms were evaluated with the Beck's Depression Inventory II (BDI‐II) [22, 23], the Generalised Anxiety Disorder 7‐Item Scale (GAD‐7) [24], a modified 6‐item version of the Deliberate Self‐harm Inventory (DSHI) [10], and the Paykel Suicide Scale (PSS) [25]. Depression and anxiety were respectively defined by BDI‐II score ≥ 20 and GAD‐7 score ≥ 10, which are the cut‐offs recommended to define moderate symptoms. Self‐injury was defined as repeating any of the self‐injurious acts included in the DSHI for at least 3–4 times that is an answer ≥ 3 to any of the DSHI items. Suicide ideation was defined by having seriously considered or planned suicide during the previous 2 weeks, that is, an answer different than “never” to the 4th item of the PSS [26]. A composite “mental ill health” variable was defined by the presence of any of the above manifestations.

### 2.3 Dysmenorrhea

An ad‐hoc multiple‐choice item assessed menstrual pain's functional impact and severity. Respondents were asked if they were affected in their everyday life by menstrual pain (“*are you affected in your everyday life?*”) with four response levels: 1‐*no*; 2‐*seldom*; 3‐*yes, but I can cope with it*; and 4‐*yes, and I'm having a hard time coping with it*. The item was derived from a previous multidimensional scoring system for dysmenorrhea which had good correlation with a linear analogue scale in earlier Swedish studies [3, 27]. For the purpose of this study, levels 3 and 4 defined dysmenorrhea (i.e., menstrual pain affecting everyday life), whereas level 4 was conceptualised as severe dysmenorrhea (i.e., dysmenorrhea that is hard to cope with).

### 2.4 Statistical Analysis

Differences in the frequencies of dysmenorrhea and severe dysmenorrhea between groups with or without mental ill health manifestations were assessed with Pearson's Chi‐squared test. Associations between mental health and dysmenorrhea were further studied with logistic regression derived odds ratios (OR). Log‐link binomial regression analyses were performed to estimate prevalence ratios (PR) for each mental ill health manifestation. Separate sets of analyses were conducted considering either dysmenorrhea or severe dysmenorrhea as the response variable. Multivariable analyses were carried out to account for the effect of potential confounders including age (years, continuous), country of birth (Sweden/other), age of menarche (years, continuous), and hormonal contraception (yes/no). Missing values, which were minimal in the analysed dataset (Table 1), were handled using listwise deletion. Sensitivity analyses using Firth's penalised logistic regression were conducted to assess the findings' robustness and potential bias due to small sample sizes. The results of these regression analyses were plotted as crude and adjusted odds ratios and prevalence ratios with 95% confidence intervals (CI). Differences were considered statistically significant at a *p*‐value < 0.05. The analyses were carried out in R with RStudio v.2023.6.0.421 for macOS [28, 29].

**TABLE 1:** Characteristics of the study population.

Characteristic (*N* = 1054)	Mean or *n*	SD or %
Age	14.1	0.7
Born in Sweden	955	90.9%
*(missing)*	(3)	
Age of menarche	12.2	1.0
Hormonal contraception	41	3.9%
*(missing)*	(2)	
Mental ill health (any)	366	34.7%
Depression	186	17.6%
Anxiety	202	19.2%
Self‐injury	161	15.3%
Suicide ideation	146	13.9%
Dysmenorrhea	581	55.1%
Severe dysmenorrhea	123	11.7%

### 2.5 Patient and Public Involvement

The YAM school‐based project was run in collaboration between researchers at Karolinska Institutet and Stockholm Region, which is the people‐elected local authority responsible for healthcare services. Throughout the project, coordination between researchers and responsible persons in participating schools (e.g., headmasters) was in place. Furthermore, on‐site meetings between researchers and school staff and/or parents were organised to share information and feedback on the project.

## 3 Results

**FIGURE 1:**
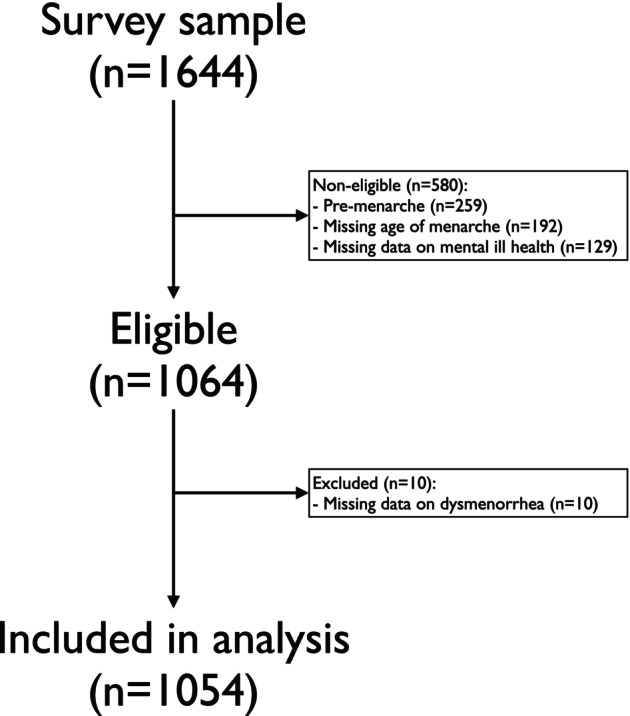
Study flow‐chart.

Of 1644 schoolgirls who were randomly sampled for the questionnaire, 1193 were eligible and 1054 of them (88.3%) were eventually included in the study (Figure 1). The mean age and age of menarche were 14.1 (SD 0.7) and 12.2 (SD 1.0), respectively (Table 1). Most girls (90.9%) were born in Sweden, and 3.9% reported hormonal contraception (Table 1).

Based on the criteria defined in the methods, 34.7% of the sample had at least one of the considered mental ill health manifestations (Table 1). The proportions of girls with depression, anxiety, self‐injury, and suicidal ideation were 17.6%, 19.2%, 15.3%, and 13.9%, respectively. Girls with mental ill health had a significantly lower age of menarche (mean difference −0.174 years; 95% CI −0.296, −0.0514) and reported hormonal contraception more frequently (6.8% vs. 2.3%, *p* < 0.001) compared to those without.

**FIGURE 2:**
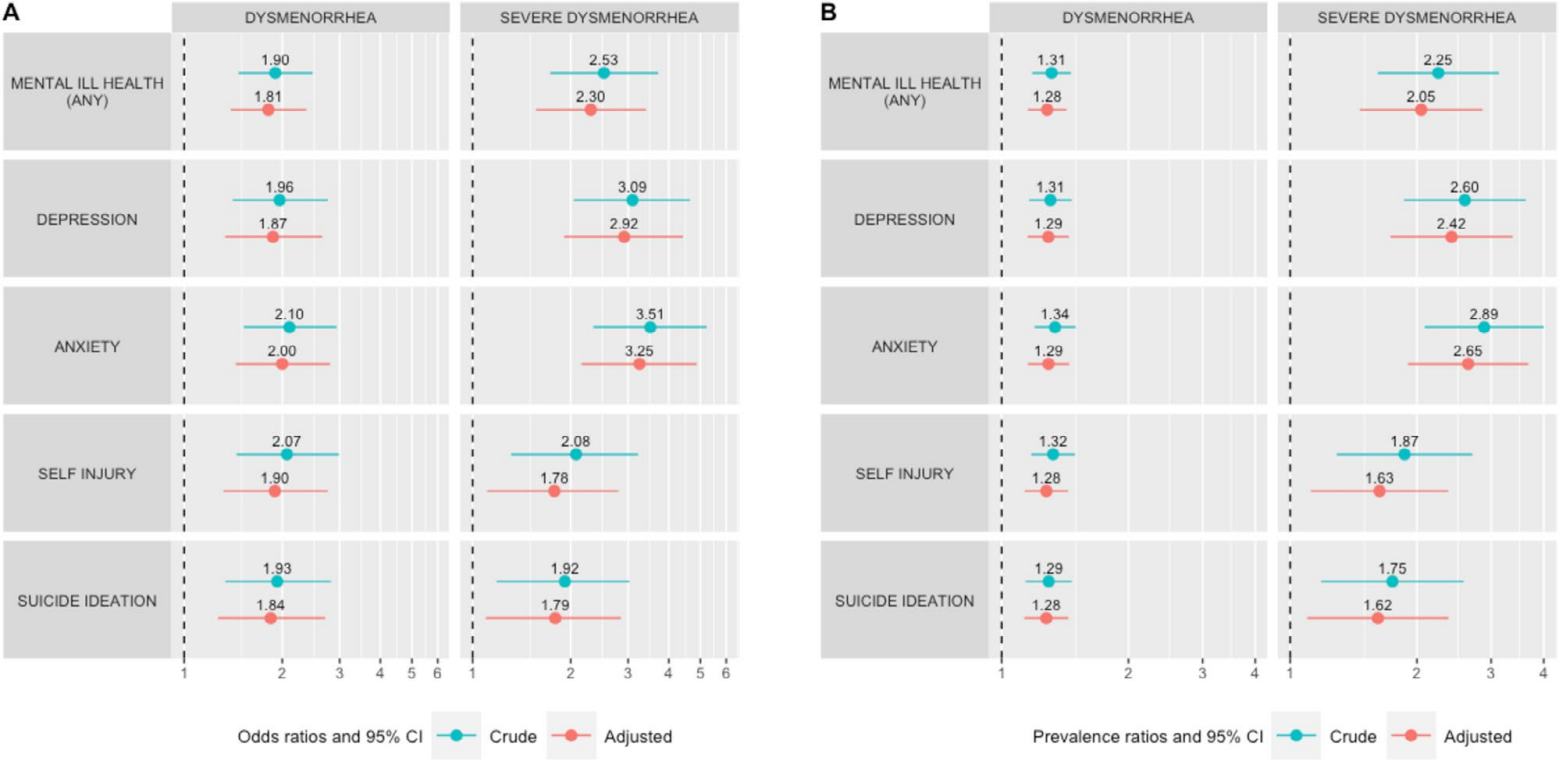
Odds ratios (A) and prevalence ratios (B) of dysmenorrhea and severe dysmenorrhea for adolescent girls with mental ill health manifestations, in relation to peers without the same manifestations. Adjusted ratios were obtained in multivariable analyses accounting for the effect of age, age of menarche, birth country, and hormonal contraception.

Overall, 581 girls (55.1%) reported dysmenorrhea, while 123 of them (11.7%) reported severe dysmenorrhea (Table 1). Both dysmenorrhea and severe dysmenorrhea were significantly associated with all the considered psychological symptoms (Table 2, Figure 2). The odds and prevalence of dysmenorrhea increased respectively by 90%–110% and 29%–34% for girls with mental ill health compared to those without (Figure 2). Similarly, severe dysmenorrhea was significantly more frequent among girls with any mental ill health manifestation (PR 2.25; 95% CI 1.61, 3.13) and more specifically with depression (PR 2.60; 95% CI 1.86, 3.63), anxiety (PR 2.89; 95% CI 2.09, 4.00), self‐injury (PR 1.87; 95% CI 1.29, 2.71), and suicide ideation (PR 1.75; 95% CI 1.18, 2.58) compared to girls without the same manifestations (Table 2, Figure 2). The above findings were stable after adjustments for age, age of menarche, country of birth, and use of hormonal contraception (Figure 2) as well as in sensitivity analyses using Firth's penalised logistic regression (Table S1).

**TABLE 2:** Prevalence of dysmenorrhea and severe dysmenorrhea among adolescent girls with and without mental ill health manifestations. All associations were tested with Pearson's Chi‐squared test and were significant at *p* < 0.001 except for severe dysmenorrhea/self‐injury (*p* = 0.001) and severe dysmenorrhea/suicide ideation (*p* = 0.006).

	Mental ill health (any)		Depression		Anxiety		Self‐injury		Suicide ideation	
	No *N* = 688	Yes *N* = 366	No *N* = 868	Yes *N* = 186	No *N* = 852	Yes *N* = 202	No *N* = 893	Yes *N* = 161	No *N* = 908	Yes *N* = 146
Dysmenorrhea										
No	346 (50.3%)	127 (34.7%)	414 (47.7%)	59 (31.7%)	411 (48.2%)	62 (30.7%)	424 (47.5%)	49 (30.4%)	427 (47.0%)	46 (31.5%)
Yes	342 (49.7%)	239 (65.3%)	454 (52.3%)	127 (68.3%)	441 (51.8%)	140 (69.3%)	469 (52.5%)	112 (69.6%)	481 (53.0%)	100 (68.5%)
Severe dysmenorrhea										
No	632 (91.9%)	299 (81.7%)	789 (90.9%)	142 (76.3%)	779 (91.4%)	152 (75.2%)	801 (89.7%)	130 (80.7%)	812 (89.4%)	119 (81.5%)
Yes	56 (8.1%)	67 (18.3%)	79 (9.1%)	44 (23.7%)	73 (8.6%)	50 (24.8%)	92 (10.3%)	31 (19.3%)	96 (10.6%)	27 (18.5%)

## 4 Discussion

### 4.1 Main Findings

In a large, random, school‐based sample of Swedish postmenarchal girls, dysmenorrhea was significantly associated with mental ill health manifestations such as depression, anxiety, self‐injury, and suicide ideation. The associations were particularly strong for severe dysmenorrhea, whose prevalence was significantly higher among girls with depression (PR 2.60), anxiety (PR 2.89), self‐injury (PR 1.87) and suicide ideation (PR 1.75) compared to peers without the same manifestations. The study findings were consistent when accounting for the effect of age, age of menarche, country of birth, and use of hormonal contraception in multivariable analyses.

### 4.2 Strengths and Limitations

This is one of the few studies addressing dysmenorrhea in a particularly vulnerable group such as postmenarchal adolescents with mental ill health [17]. A high response rate from a large population‐based sample mitigates selection bias compared to clinical samples or selected sub‐populations [4]. A broad range of psychological symptoms was measured with internationally validated instruments. Dysmenorrhea was categorised according to the person‐reported impact of menstrual pain on everyday life, and separate analyses were conducted for overall and severe dysmenorrhea. The potential effect of covariates such as age, age of menarche, and hormonal contraception was accounted for in multivariable analyses.

There are however important limitations to consider. The directionality of the associations cannot be verified because of the cross‐sectional design. The variables of interest were self‐reported and not defined by clinical diagnoses. Elevated levels of psychological symptoms, as detected by self‐report questionnaires, are indeed suggestive of mental health conditions, but precise diagnoses would require semi‐structured interviews [18], which were unfeasible in the context of this study. For similar reasons, no distinction could be made between primary and secondary dysmenorrhea. Although most cases among adolescents are primary, some stem from underlying conditions (e.g., endometriosis) which can specifically affect the association with mental health, for example because of more severe or complex symptoms or through psychosocial mechanisms [7, 30, 31, 32]. It should be considered that not all covariates may be investigated through self‐reporting. Therefore, despite the mentioned adjustments, residual confounding from unmeasured factors is possible. Additionally, there were relatively few severe dysmenorrhea cases in some subgroups (e.g., those reporting self‐injury or suicidal ideation). In such cases, adjusting for multiple confounders may require larger samples to reduce bias and improve precision.

### 4.3 Interpretation

Adolescence is a period of intense biological, psychological, and social development, during which young women are exposed to peculiar vulnerabilities. Postpubertal girls develop psychological symptoms to a greater extent than their male peers, and many of them start suffering from menstrual pain soon after menarche. The findings of a recent systematic review [17] highlight that only a few studies have so far investigated the relationship between mental health and dysmenorrhea, particularly among adolescents. Associations have been observed in previous samples proceeding from diverse populations and regions, but most of the studies have so far focused on depression and anxiety symptoms in adult samples [17]. Even fewer studies have investigated the association of self‐injury or suicidality with adolescent dysmenorrhea and, more generally, chronic pain during early postmenarchal years [17, 33]. In a Chinese sample (mean age 15.0; range 12–18), non‐suicidal self‐injury was significantly more frequent among girls with mild (OR 1.31), moderate (OR 1.65), or severe (OR 2.17) dysmenorrhea compared to girls without these [34]. In a Swiss study, significantly more adolescents (age 16–20) with severe dysmenorrhea reported past suicide attempts compared to peers with no, mild, or moderate dysmenorrhea (5.6% vs. 3.1%) [35].

Our present findings add evidence of a strong association between mental health and dysmenorrhea among postmenarchal adolescents. Such associations are likely to be the result of complex bidirectional mechanisms. Underlying mental health conditions, such as depression and anxiety, may amplify pain perception, leading to worsened menstrual pain.

Mental ill health can indeed precede a variety of physical symptoms, among which pain is the most common [36, 37]. Longitudinal studies have identified psychological symptoms and disorders among adolescents as risk factors for both pain and menstrual symptoms [38, 39]. Co‐occurrence can also be explained by interrelated vulnerabilities of genetic, neurobiological, and psychosocial nature [18, 36, 37, 40].

Chronic pain can, in turn, exacerbate psychological distress and contribute to anxiety, depression, and even suicidal ideation. The impact of dysmenorrhea on physical and psychosocial wellbeing can be particularly intense due to the normalisation, stigma, and inadequate healthcare responses that affect adolescent menstrual health [41, 42]. Pain‐related social withdrawal can, for instance, induce or worsen psychological distress linking dysmenorrhea with more serious psychopathological outcomes [43]. At the same time, it has been observed that the excess of pain‐related psychological distress only partially explains outcomes such as self‐injury and suicidal ideation in association with chronic pain conditions [33, 43].

Therefore, although unpreventable physiologic processes, such as changes in sex hormones, may explain both physical (e.g., dysmenorrhea) and psychological (e.g., depression or anxiety) symptoms during adolescence, the ultimate impact on health and well‐being likely depends on complex interactions between biological, psychological, and social factors [14, 16, 41, 44, 45]. This is arguably consistent with biopsychosocial frameworks according to which different domains (i.e., biological, psychological, and social) are relevant to conditions such as chronic pain or dysmenorrhea, although their relative contribution may have large individual variations [31, 45, 46]. The fear‐avoidance model identifies catastrophising and fear of pain as key drivers of vicious circles linking pain and mental health through effects on both pain severity and decline in psychological functioning [18, 47]. Our finding of a particularly strong association in the case of severe dysmenorrhea, for example, could suggest that psychological difficulties interfere with the adaptation process required to cope with menstrual pain and that the resulting maladjustment worsens pain perception and related well‐being [48, 49].

While further studies and longitudinal data are clearly needed, the conceptualisation of complex pathways, including individual or socio‐contextual aggravating/mitigating factors, seems important to understand dysmenorrhea and to inform personalised interventions [36, 37, 46]. At the same time, we believe that cross‐domain outcomes in adolescent mental and menstrual health research (e.g., physical pain or psychological wellbeing, respectively) should be considered for inclusion in future core outcome sets for interventional studies [50, 51].

## 5 Conclusions

This study provides evidence of a strong association between dysmenorrhea and several manifestations of mental ill health, such as depression, anxiety, self‐injury, and suicidal ideation, among postmenarchal adolescents. These associations are likely to be the result of complex bidirectional mechanisms. Dysmenorrhea is often overlooked in adolescents, and its reporting and management can be even more challenging among those with psychological difficulties.

These findings highlight the need for increased vigilance in school‐ and community‐based healthcare settings, where opportunistic screening may be beneficial. Healthcare providers should consider the possibility of concurrent menstrual and psychological symptoms during adolescent health visits to facilitate early identification. Additionally, multidisciplinary collaboration is crucial in addressing the needs of adolescents with both dysmenorrhea and mental health challenges. Future research should focus on improving our understanding of the biopsychosocial factors involved and exploring novel integrated interventions.

## Author Contributions

P.G.: conceptualisation; methodology; investigation; resources; formal analysis; writing – original draft; and review and editing; lead and corresponding author. G.H.: methodology; investigation; and resources; YAM: co‐investigator; writing – review and editing. D.W.: investigation and resources; YAM: principal investigator; writing – review; and editing. V.C.: methodology; investigation; data curation; resources; and YAM: co‐investigator; writing – review and editing.

## Ethics Statement

The school‐based YAM project was approved by Stockholm's regional ethical review board (Regionala Etikprövningsnämnden i Stockholm; 2015/2175‐31/5). Participation was voluntary and required informed consent of pupils and caregivers (for pupils younger than 15). Only anonymous data were analysed in the study.

## Conflicts of Interest

The authors declare no conflicts of interest.

## Supporting information


**Table S1.** Results of logistic regression analyses.

## Data Availability

The data that support the findings of this study are available on request from the corresponding author. The data are not publicly available due to privacy or ethical restrictions.
