# Supplementary material for: Dysmenorrhea and Adolescent Mental Health: A School‐Based Cross‐Sectional Study
Source: BJOG. 2025 Apr 24;132(9):1278–84. doi: 10.1111/1471-0528.18187 (PMC12232528; doi:10.1111/1471-0528.18187)
Supplement: Supplementary file 1 — Table S1. Results of logistic regression analyses. [file BJO-132-1278-s001.pdf]

Table S1. Results of logistic regression analyses

Dependent_Variable	Independent_Variable	GLM_Crude	Firth_Crude	GLM_Adjusted	Firth_Adjusted
Dysmenorrhea	Mental ill health (any)	1.90 [1.47, 2.48]	1.90 [1.46, 2.47]	1.81 [1.39, 2.37]	1.81 [1.39, 2.36]
Dysmenorrhea	Depression	1.96 [1.41, 2.76]	1.95 [1.40, 2.75]	1.87 [1.33, 2.65]	1.86 [1.33, 2.63]
Dysmenorrhea	Anxiety	2.10 [1.52, 2.94]	2.10 [1.52, 2.92]	2.00 [1.44, 2.80]	1.99 [1.43, 2.78]
Dysmenorrhea	Self injury	2.07 [1.45, 2.98]	2.05 [1.44, 2.96]	1.90 [1.32, 2.76]	1.88 [1.31, 2.73]
Dysmenorrhea	Suicide ideation	1.93 [1.34, 2.82]	1.92 [1.33, 2.80]	1.84 [1.27, 2.71]	1.83 [1.26, 2.68]
Severe Dysmenorrhea	Mental ill health (any)	2.53 [1.73, 3.71]	2.52 [1.73, 3.70]	2.30 [1.56, 3.40]	2.29 [1.56, 3.37]
Severe Dysmenorrhea	Depression	3.09 [2.04, 4.64]	3.10 [2.05, 4.65]	2.92 [1.91, 4.42]	2.91 [1.91, 4.39]
Severe Dysmenorrhea	Anxiety	3.51 [2.35, 5.22]	3.51 [2.35, 5.22]	3.25 [2.16, 4.87]	3.23 [2.15, 4.83]
Severe Dysmenorrhea	Self injury	2.08 [1.31, 3.22]	2.09 [1.33, 3.23]	1.78 [1.11, 2.80]	1.79 [1.12, 2.80]
Severe Dysmenorrhea	Suicide ideation	1.92 [1.18, 3.03]	1.94 [1.20, 3.05]	1.79 [1.10, 2.85]	1.80 [1.11, 2.86]

Values are odds ratios [95% confidence interval], crude or adjusted for age, age of menarche, country of birth and use of hormonal contraception.
